# Supplementary material for: Mapping the global design space of nanophotonic components using machine learning pattern recognition
Source: Nat Commun. 2019 Oct 21;10:4775. doi: 10.1038/s41467-019-12698-1 (PMC6803653; doi:10.1038/s41467-019-12698-1)
Supplement: Supplementary file 1 — Supplementary Information [file 41467_2019_12698_MOESM1_ESM.pdf]

Mapping the global design space of nanophotonic components using machine learning pattern recognition

- Supplementary information -

Daniele Melati[1,†], Yuri Grinberg[2,†], Mohsen Kamandar Dezfouli[1], Siegfried Janz[1], Pavel Cheben[1], Jens H. Schmid[1], Alejandro Sánchez-Postigo[3] and Dan-Xia Xu[1,*]

[1]*Advanced Electronics and Photonics Research Centre, National Research Council Canada, 1200 Montreal Rd., Ottawa, ON K1A 0R6, Canada*
[2]*Digital Technologies Research Centre, National Research Council Canada, 1200 Montreal Rd., Ottawa, ON K1A 0R6, Canada*
[3]*Universidad de Málaga, Departamento de Ingeniería de Comunicaciones, ETSI Telecomunicación, Campus de Teatinos s/n, 29071 Málaga, Spain*

*e-mail: dan-xia.xu@nrc-cnrc.gc.ca
[†]These authors contributed equally to this work.

Supplementary Note 1: PCA error analysis

As defined in the manuscript, for the first grating structure (Fig. 2 in the Manuscript) we quantify the "difference" between two different designs A and B using the Manhattan distance

$\sum_{i=1}^{5} |L_{\mathrm{i,A}} - L_{\mathrm{i,B}}|$.

Error analysis is conducted by quantifying the distance between a good design in the original parameter space found by the global optimizer and the nearest corresponding projection on the 2-D hyperplane. Our procedure to compute this approximation error goes as follow:

a. we sample randomly *N* good designs (with *N*=3 as minimum) within the pool of *M* good designs generated in stage 1 (*M* = 45 for the structure in Fig. 2);
b. with this subset we compute the hyperplane through PCA;
c. we compute the average error in terms of the Manhattan distance from the hyperplane to the remaining *M*-*N* designs;
d. we compute the worst error as the largest Manhattan distance from the hyperplane to all the designs;
e. we repeat (a)-(d) 1000 times random sampling *N* good designs and we take the empirical expectation for both worst case and average error.

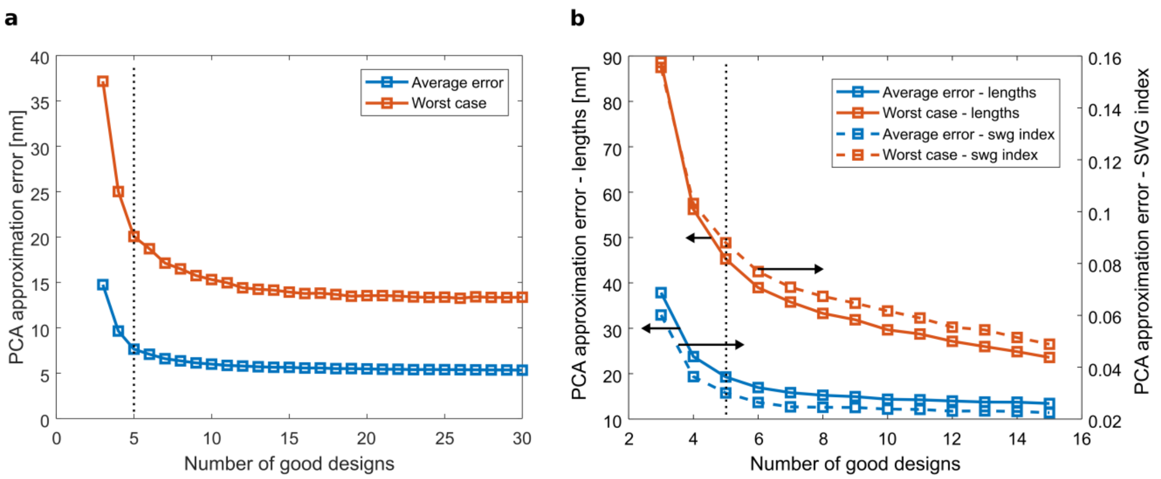

Supplementary Figure 1 Error analysis. Error introduced by approximating the pool of good design found in stage 1 with the nearest corresponding design on the 2-D hyperplane found by PCA for (**a**) first grating structure (Fig. 2 in the Manuscript) and (**b**) the grating structure incorporating the subwavelength (SWG) metamaterial (Fig. 5 in the Manuscript). For the first case using more than 5 designs does not significantly reduce the approximation error. For the grating incorporating the metamaterial the error contributions are split between segment lengths and effective material index n_{swg}. Also in this case 5 good designs are sufficient to obtain a good approximation accuracy.

The obtained numbers hence represent the expected average and expected worst case Manhattan errors under the random sampling of the corresponding N designs from the dataset. Results are reported in the Supplementary Figure 1a.

Using more than 5 good designs does not significantly reduce the error, especially on average. With $N = 5$ the average error is below 8 nm and the worst case is below 20 nm. Feeding additional good designs to PCA does not significantly modify the discovered hyperplane, confirming that 5 initial good designs represent in this case a good compromise between accuracy and computational effort required in stage 1.

If we use all of the $M = 45$ good designs to compute the hyperplane then the errors must be computed using again the entire pool of 45 good designs. In this case we obtain an average error of 6 nm and a worst case below 12 nm.

For the second grating structure incorporating the subwavelength (SWG) metamaterial (Fig. 5a in the manuscript), the same procedure is applied to compute the approximation error. In this case two different distances are calculated, one for segment lengths and one for the effective material index n_{swg}. Results are reported in Supplementary Figure 1b for the average error and worst case. With 5 initial good designs we obtain an average error of 19 nm for lengths and 0.03 for refractive index. Worst cases are 45 nm and 0.09, respectively. Despite the errors being higher due to the more complex grating design, 5 designs are still enough to obtain a good accuracy. The error obtained using all 19 good designs to compute the hyperplane is on average 10 nm for segment length (Manhattan distance wise) and 0.02 for the refractive index. The worst case errors are 20 nm and 0.04, respectively

Furthermore, for the first grating structure we verify if 2 principal components are sufficient to describe all good designs. A PCA-based approximation of 45 designs to 1-D incurs an average error of more than

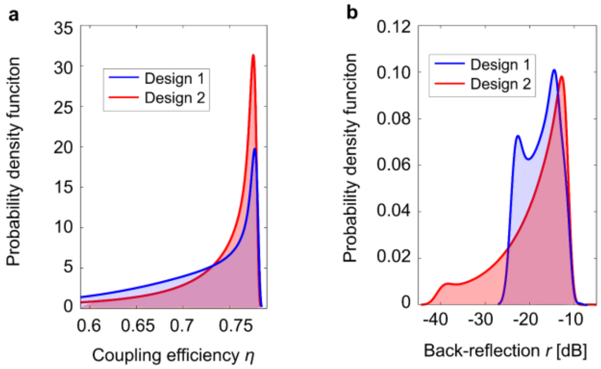

Supplementary Figure 2 Tolerance to fabrication uncertainty. Probability density functions for (**a**) coupling efficiency and (**b**) back-reflections for designs 1 and 2 described in the manuscript. Design 1 shows a longer tail towards lower values of η, slightly reducing the probability to obtain a high coupling efficiency. Design 2 has a much smaller value of back-reflection that quickly grows when width variations are introduced (longer tail of the function). On the contrary, the variability of back-reflections for design 1 is considerably smaller as demonstrated by the narrower probability density function, but a reflection less than -25 dB is never obtained.

50 nm, while a 3-D approximation incurs an average error of 3 nm. This warrants our choice of 2D approximation as the error reduction by adding the second dimension is significant, while further addition of a third dimension leads to a very small improvement. The same analysis is conducted for the second grating structure leading to the same conclusion.

Supplementary Note 2: Uncertainty analysis with polynomial chaos expansion

In order to verify the results of the uncertainty analysis for width deviations described in the manuscript, we compute the probability density functions of the coupling efficiency and back-reflections for designs 1 and 2 (see Fig. 3 in the manuscript). Width deviations δ_w are assumed to be normally distributed with zero mean and a standard deviation of 5 nm[1]. Probability density functions are efficiently calculated using 2D-FDTD in combination with a polynomial chaos model[2–4]. Eighty different values for δ_w are sampled according to their distribution. Corresponding designs are generated and simulated with 2D-FDTD to obtain the coupling efficiency and back-reflection for each of them. For both quantities a corresponding stochastic surrogate model describing their dependence on δ_w (polynomial chaos model) is realized with fifteenth-order Hermite polynomials as the orthonormal basis. The coefficients of the polynomials are estimated from the 80 simulations with a compressed sensing technique solving a corresponding basis pursuit denoise problem with the freely available spgl1 solver[5]. Details on how to compute the polynomial chaos surrogate model can be found in ref 4. Finally, the two probability density functions are obtained with a standard Monte Carlo simulation by sampling the surrogate models 5000 times (which only takes few seconds) and using a Gaussian kernel density estimator.

Although the polynomial chaos model allows to accurately compute the required stochastic properties with a limited number of 2D-FDTD simulations, the previous analysis based on PCA is of fundamental importance to first identify the possible design candidates deserving further analysis. Regarding coupling efficiency (Supplementary Figure 2a), the probability density functions of both designs are right-bounded

by the value obtained without considering uncertainty (about 0.76 in both cases) but design 1 shows a longer tail towards lower values of η. This means that the probability to obtain a high coupling efficiency is lower compared to design 2 and therefore a lower fabrication yield is expected for design 1. As shown in Supplementary Figure 2b, also the lower value of the two back-reflection probability density functions is limited by the performance obtained without uncertainty. As discussed in the manuscript, without uncertainty design 2 has substantially smaller back-reflections (smaller than -37 dB) but as predicted by the map of Fig. 4b in the manuscript the region of low back-reflection is highly localized, and back-reflection quickly grows when width variations are introduced (longer tail of the function). In contrast, the minimum back-reflection achievable by design 1 is much higher but its variability is considerably smaller, as demonstrated by the narrower probability density function. In both cases back-reflections do not exceed -10 dB (worst-case-scenario) with the considered uncertainty.

Supplementary Note 3: Additional information on the subwavelength gratings

As we have mentioned in the main text, four segment gratings with transverse subwavelength engineering can be still simulated using a 2D Maxwell solver, where the subwavelength segment is replaced by an effective medium with effective material index n_{swg}. The value of n_{swg} can be modified between that of silica and silicon by adjusting the pitch and the duty cycle of the subwavelength patterning in the transverse direction. To achieve the transverse 100 nm feature size in compatibility with deep UV lithography, the allowable metamaterial index is found to be $1.67 < n_{swg} < 2.86$ if one uses a subwavelength segmentation period of 450 nm[6].

The details of the three selected designs marked in Fig. 5 of the manuscript are presented in Supplementary Table 1. This includes the coordinates on the reduced 2D hyperplane, the corresponding structural parameters, as well as the performance figures of merit. As can be seen, the three devices are geometrically quite distinct (quantified using the Manhattan distance), however they all have similar performance. Note that all of the four lengths involved are larger than 100 nm for the three designs. Additionally, all three devices have an effective material index within the feasible range mentioned above. Therefore 100 nm feature size in both directions is ensured.

Supplementary Table 1: Structural and performance parameters of selected swg-based gratings marked in Fig. 5.

Design	$[\alpha, \beta]$	$L_1, \cdots, L_4$ [nm]	n_{swg}	Λ [nm]	Distance [nm]	η	r (dB)	BW (nm)
**1**	[-0.93, 2.06]	270, 103, 216, 144	2.50	733	-	0.74	-21	45.6
**2**	[0.29, 2.54]	289, 106, 233, 121	2.43	749	62	0.74	-20	47.0
**3**	[1.32, 2.44]	302, 101, 251, 101	2.41	755	112	0.75	-24	48.0

Distance refers to the Manhattan distance with respect to design 1, computed considering only the segment lengths. The coupling efficiency η and reflection r refer to the values at a wavelength of 1550 nm.

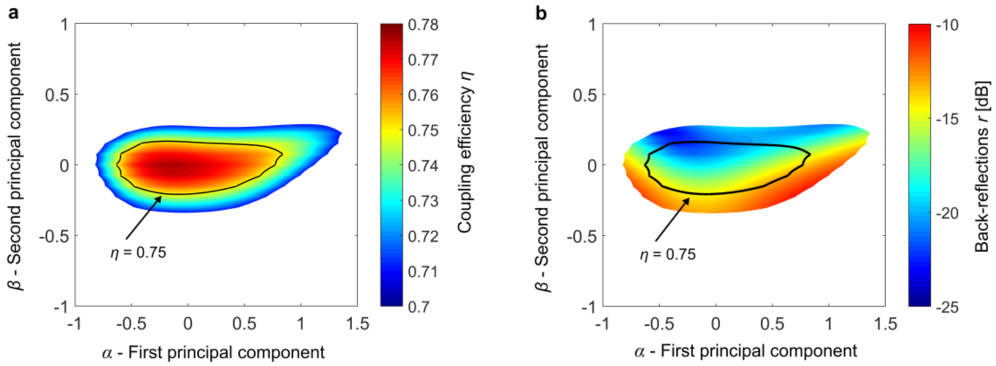

Supplementary Figure 3 Design of vertical grating couplers at λ = 1310 nm. The two maps show the coupling efficiency (**a**) and back-reflection (**b**) across the sub-space of good designs for a grating with the same structure shown in Fig. 2a in the manuscript but operating at λ = 1310 nm. PCA allows again to efficiently representing the design space using a 2-D hyperplane.

Supplementary Note 4: Generality of hyperplanes: vertical grating couplers for the optical communication O-band

The hyperplanes described in the manuscript provide a comprehensive characterization of the grating design space for C band. The optical communication O-band (1260 nm to 1360 nm) is another important wavelength range particularly for data centers[7]. We thus apply dimensionality reduction to design vertical grating couplers at a wavelength λ = 1310 nm, using the same grating structure shown in Fig. 2 in the manuscript. Material indices (3.50 and 1.45 for silicon and silica, respectively) and SMF-28 fiber mode (MFD 9.2 μm) are adjusted for the reduced wavelength. We execute the optimization stage 1 until it generates 5 different designs with a coupling efficiency of η > 0.75. The highest value was found to be η = 0.77. Following the analysis for λ = 1550 nm, 5 designs are speculated to be sufficient to define the reduced parameter space through PCA as long as the approximation errors are small. Indeed, our results confirm that the sub-space of high performance designs can be accurately represented on a 2-D hyperplane incurring an average approximation error smaller than 2 nm.

The vectors defining the α-β hyperplane are $\mathbf{V}_{1\alpha\beta}$ = [11.50, -7.76, 32.60, 22.59, -28.46] nm, $\mathbf{V}_{2\alpha\beta}$ = [11.18, 3.91, 34.07, -52.56, 0.76] nm and $\mathbf{C}_{\alpha\beta}$ = [59.40, 63.2, 50, 105.6, 238] nm (see equation (2) in the manuscript).

Supplementary Figures 3a and 3b report the results of the exhaustive exploration of the sub-space on the α-β hyperplane limited to the region defined by η > 0.70. The same unit (100 nm per division) in α or β is used as in Figs. 3a and 3b in the manuscript. Identifying the region of top performing designs through an exhaustive brute force search in a five-dimensional space would incur an increase of several orders of magnitude in computation time, similarly to the λ = 1550 nm case study.

Considering that the basic grating structure is unchanged, the sub-space of good designs looks remarkably different compared to that found for λ = 1550 nm. For the latter, good designs occupied a sub-space with approximately the same size in α and β (about 3 units, Figs. 3a and 3b in the manuscript). In the case for λ

= 1310 nm the range allowed on α is similar (about 2 units) while a tighter choice is available along β (about 0.6 units). Nonetheless a design area with $\eta > 0.75$ can still be identified with designs ensuring similar fiber coupling efficiency but different back-reflections, ranging from -22 dB to -13 dB. Within this area the minimum feature size ranges between 30 nm and 60 nm, depending on the selected design.

Supplementary References

1. Xu, D.-X. *et al.* Silicon Photonic Integration Platform—Have We Found the Sweet Spot? *IEEE J. Sel. Top. Quantum Electron.* **20**, 189–205 (2014).
2. Xiu, D. Fast Numerical Methods for Stochastic Computations: A Review. *Commun Comput Phys* 31 (2009).
3. Weng, T.-W., Melati, D., Melloni, A. & Daniel, L. Stochastic simulation and robust design optimization of integrated photonic filters. *Nanophotonics* **6**, 299–308 (2017).
4. Melati, D. *et al.* Performance robustness analysis in machine-assisted design of photonic devices. in *Smart Photonic and Optoelectronic Integrated Circuits XXI* **10922**, 1092203 (International Society for Optics and Photonics, 2019).
5. van den Berg, E. & Friedlander, M. Probing the Pareto Frontier for Basis Pursuit Solutions. *SIAM J. Sci. Comput.* **31**, 890–912 (2008).
6. Cheben, P. *et al.* Refractive index engineering with subwavelength gratings for efficient microphotonic couplers and planar waveguide multiplexers. *Opt. Lett.* **35**, 2526–2528 (2010).
7. Effenberger, F. J. Industrial Trends and Roadmap of Access. *J. Light. Technol.* **35**, 1142–1146 (2017).
